# CD4+ T cell-mimicking nanoparticles encapsulating DIABLO/SMAC mimetics broadly neutralize HIV-1 and selectively kill HIV-1-infected cells

**DOI:** 10.7150/thno.59728

**Published:** 2021-08-25

**Authors:** Grant R. Campbell, Jia Zhuang, Gang Zhang, Igor Landa, Luke J. Kubiatowicz, Diana Dehaini, Ronnie H. Fang, Liangfang Zhang, Stephen A. Spector

**Affiliations:** 1 Division of Infectious Diseases, Department of Pediatrics, University of California San Diego, La Jolla, California, USA.; 2 Department of Nanoengineering, University of California San Diego, La Jolla, California, United States of America.; 3 Moores Cancer Center, University of California San Diego, La Jolla, California, United States of America.; 4 Rady Children's Hospital, San Diego, California, United States of America.

**Keywords:** HIV, nanoparticle, SMAC mimetics, autophagy, neutralization

## Abstract

HIV-1 is a major global health challenge. The development of an effective vaccine and a therapeutic cure are top priorities. The creation of vaccines that focus an antibody response toward a particular epitope of a protein has shown promise, but the genetic diversity of HIV-1 stymies this progress. Therapeutic strategies that provide effective and broad‐spectrum neutralization against HIV-1 infection are highly desirable.

**Methods:** We investigated the potential of nanoengineered CD4+ T cell membrane-coated nanoparticles (TNP) encapsulating the DIABLO/SMAC mimetics LCL-161 or AT-406 (also known as SM-406 or Debio 1143) to both neutralize HIV-1 and selectively kill HIV-1-infected resting CD4+ T cells and macrophages.

**Results:** DIABLO/SMAC mimetic-loaded TNP displayed outstanding neutralizing breadth and potency, and selectively kill HIV-1-infected cells via autophagy-dependent apoptosis while having no drug-induced off-target or cytotoxic effects on bystander cells. Genetic inhibition of early stages of autophagy abolishes this effect.

**Conclusion:** DIABLO/SMAC mimetic loaded TNP have the potential to be used as therapeutic agents to neutralize cell-free HIV-1 and to kill specifically HIV-1-infected cells as part of an HIV-1 cure strategy.

## Introduction

Although antiretroviral therapy (ART) has greatly improved life expectancy and quality for those infected with human immunodeficiency virus type 1 (HIV-1), multi-drug resistance continues to increase, and over 38 million people remain chronically infected. Current efforts to purge the latent HIV-1 reservoir strive to induce transcription of latent HIV-1 proviruses using latency-reversing agents (LRAs) followed by a combination of ART, targeted immunotherapy, host immune clearance, and HIV-1-cytolysis to kill latently infected cells - the “kick and kill” strategy. However, with over 160 latency reactivation compounds tested to date, none have shown great promise [reviewed in 1]. The continued absence of both a potential vaccine and a functional cure underscores the need for innovative approaches in their development.

During HIV-1 infection, the transcriptome and proteome of infected macrophages is substantially altered rendering them resistant to the cytopathic effects of infection and to CD8+ T cell-mediated killing 2-4. In both CD4+ T cells and macrophages, HIV-1 increases the expression of anti-apoptosis proteins including the inhibitor of apoptosis proteins (IAP) X-linked inhibitor of apoptosis (XIAP), baculoviral IAP repeat containing (BIRC) 2 (umquhile cIAP1), and BIRC3, as well as BCL2 family members, while decreasing the expression of pro-apoptotic proteins 5-12. The overexpression of IAPs can inhibit cell death through the direct antagonism, ubiquitination, and/or neddylation of apoptosis executioner caspases and apoptosis initiator caspases 13, 14, while also inhibiting LTR-dependent HIV-1 transcription 15, 16, thus playing a potential role in the establishment and maintenance of HIV-1 latency. IAPs can also ubiquitinate receptor-interacting protein kinase (RIPK) 1, a death domain containing protein that controls differentiation and inflammation transcriptional responses as well as the assembly of apoptosis and necroptosis signaling platforms 17. Consequently, IAPs are excellent targets for therapeutic exploitation in HIV-1 cure strategies.

The deregulated apoptosis caused by overexpression of IAPs in oncogenesis led to the identification of DIABLO/SMAC peptidomimetics (SM). These SM mimic the BIR-binding N-terminal tetrapeptide sequence of DIABLO, a proapoptogenic mitochondrial protein that is released into the cytoplasm in response to apoptotic stimuli and antagonizes IAPs. The interaction of SM with BIRC2 and BIRC3 activates their E3 ubiquitin ligase activity promoting their autoubiquitination and proteasomal degradation 18-20, while SM-XIAP binding leads to disinhibition of caspases 21. Under conditions of stress, this results in cell death. We previously demonstrated that SM treatment of HIV-1-infected macrophages (HV-Mφ) and HIV-1-infected resting memory CD4+ T cells (HIV-T_CM_) induced XIAP and BIRC2 degradation leading to the induction of macroautophagy (hereafter referred to as autophagy) and apoptosis of HIV-1-infected cells but not bystander cells through the selective assembly of a death-inducing signaling complex on phagophore membranes in HIV-1-infected cells that includes both autophagy ([autophagy related [ATG] 5, ATG7 and the polyubiquitin-binding protein sequestosome 1 [SQSTM1, p62]) and pro-death (FADD, RIPK1, RIPK3, caspase 8) proteins.

The emergence of nanomedicine has provided new avenues for HIV-1 treatment and prevention research. Nanoparticles are being assessed as vehicles for antiviral drugs to improve drug tolerability, circulation half‐life, efficacy, and as carriers for delivery to the central nervous system 22-27. They are also being evaluated to deliver small interfering RNAs (siRNAs) to silence gene expression in infected cells, as well as HIV-1 itself [reviewed in 28], and to deliver cargo(es) that directly interfere with and inhibit viral replication 25, 29 or to selectively kill HIV-1-infected cells 30. The targeted delivery of nanoparticles to specific cells or anatomic sites can be achieved through the conjugation of cell-specific ligands to the nanoparticle surface. However, this process requires protein identification and labor-intensive synthesis, and can result in the destabilization of the nanoparticle or in the ligand losing biological activity due to interactions with the nanoparticle surface. These issues can be bypassed by using cell membrane-coated nanoparticles, made by wrapping the membranes of mammalian cells onto synthetic nanoparticle cores, thus using natural cell membranes and their receptors for biointerfacing. The unique properties of these cell membrane-coated nanoparticles have resulted in them being evaluated as a biomimetic platform to treat a number of diseases 31-38 and led us to investigate their potential use against HIV-1 39, 40. We previously demonstrated that uninfected CD4+ T cell plasma membrane wrapped poly(lactic‐*co*‐glycolic acid) (PLGA) core nanoparticles (TNP) mimic CD4+ T cells 39 and neutralize every virus in a global multi-subtype 125-virus panel with a geometric mean IC_80_ of 819 *μ*g/mL (range 72-8570 *µ*g/mL). These TNP specifically targeted HIV-1-infected cells and inhibited viral release while reducing cell-associated HIV-1 in a dose- and autophagy-dependent manner with no deleterious effects observed in uninfected bystander cells 40. The biodegradable PLGA nanoparticle core used in these TNP is a versatile drug delivery platform that can be loaded with peptides, siRNA, CRISPR/Cas9, or small molecules. Upon cellular uptake, the degradation of PLGA enables release of the encapsulated payload, thus allowing it to exert biological activity 41, 42. In this study, we encapsulated the DIABLO/SMAC mimetics LCL-161 43 and AT-406 44 within TNP and examined their ability to neutralize HIV-1 and to kill specifically HIV-1-infected macrophages and resting memory CD4+ T cells.

## Methods

### Formulation and characterization of nanoparticles

PLGA nanoparticle cores were prepared using 0.67 dL/g carboxy-terminated 50∶50 poly(_DL_-lactide-*co*-glycolide) (LACTEL Absorbable Polymers). The polymer and drug payload (LCL-161 or AT-406; Selleck Chemicals) were dissolved separately in acetonitrile and mixed at the desired ratios to a final PLGA concentration of 10 mg/mL. The acetonitrile solution was then precipitated into an equal volume of water. Afterwards, the solution was placed under a vacuum aspirator until the organic solvent was completely removed. Unencapsulated drug was filtered out using 100 kDa MWCO Amicon ultrafiltration devices (Millipore Sigma). For membrane coating, the drug-loaded PLGA cores and cell membrane collected from SUP-T1 cells 39 were mixed together at a polymer to membrane protein weight ratio of 1:1, followed by sonication using a Fisher Scientific FS30D bath sonicator for 2 min. Size and zeta potential were measured by dynamic light scattering (DLS) using a Malvern Zetasizer Nano ZS. To visualize nanoparticle morphology, the nanoparticle sample was adsorbed onto a carbon-coated 400-mesh copper grid and stained with 1 wt% uranyl acetate (both Electron Microscopy Sciences), followed by imaging on a JEOL 1200 EX II transmission electron microscope. For the stability study, nanoparticle samples were stored in phosphate-buffered saline (PBS) at room temperature, and size was measured periodically by DLS over the course of 8 h. To quantify the drug release, aliquots of the nanoparticles were suspended in PBS at a drug concentration of 1 µM. At predetermined time-points, a subset of the nanoparticle aliquots was pelleted by centrifugation, and the released drug in the supernatant was quantified using an Agilent 1220 Infinity II LC HPLC system.

### HIV-1

The following were obtained from the NIH AIDS Reagent Program: HIV-1_NL4-3_ (pNL4-3) from Dr. Malcolm Martin 45, and HIV-1_Ba-L_ from Suzanne Gartner, Mikulas Popovic and Robert Gallo 46, 47. Virus stocks were prepared as previously described 10. HIV-1 infectivity was calculated as the 50% tissue culture infectious doses (TCID_50_) as described previously 48 and multiplicity of infection confirmed using TZM-bl (human; sex: female; RRID: CVCL_B478) cells from John C. Kappes, Xiaoyun Wu and Tranzyme Inc. 49. The global 12-virus panel 50 representing the major subtypes and circulating recombinant forms was a kind gift from Dennis R. Burton (Scripps Research Institute, San Diego, CA). Pseudoviruses were generated by co-transfection of HEK 293T/17 cells (human; sex: female; ATCC Cat# CRL-11268; RRID:CVCL_1926) with an Env-expressing plasmid and an Env-deficient genomic backbone plasmid (pSG3ΔEnv) using polyethylenimine (Sigma). Pseudoviruses were collected 48 h post-transfection and stored at -80 °C. TCID_50_ was determined in TZM-bl using Spearman-Karber analysis.

### Cell culture

Whole blood was drawn from HIV-1-seronegative healthy male and female volunteers, ages between 18 and 65 years, at UC San Diego Health Sciences using protocols approved by the Human Research Protections Program of the University of California San Diego in accordance with the requirements of the Code of Federal Regulations on the Protection of Human Subjects (45 CFR 46 and 21 CFR 50 and 56). All volunteers gave written informed consent prior to their participation, all samples were de-identified, and donors remained anonymous. Peripheral blood mononuclear cells (PBMC) were isolated from whole blood by density gradient centrifugation over Ficoll-Paque Plus (GE Healthcare). HIV-1-infected macrophages were prepared as previously described 11. Briefly, 6 × 10^6^ PBMC/mL were incubated in macrophage media (RPMI 1640 [Gibco] supplemented with 10% [vol/vol] heat-inactivated fetal bovine serum [FBS; Sigma], 2 mM L-glutamine, 0.1 mg/mL streptomycin, 100 U/mL penicillin [all Gibco], and 10 ng/mL CSF1 [Peprotech]) for 4 h, after which non-adherent cells were removed by aspiration and washed with Dulbecco's phosphate buffered saline (Gibco). Adherent cells were further incubated in macrophage media for 10 d at 37 ° C, 5% CO_2_ with media changes every 2 d, after which macrophages were infected with 0.04 multiplicity of infection (MOI) HIV-1_Ba-L_ for 10 d before use. HIV-1 infected, resting central memory CD4+ T cells (HIV-T_CM_) were prepared as previously described 10. Briefly, CD4+ T cells isolated from PBMC using the CD4+ T cell isolation kit (Miltenyi Biotec Cat# 130-096-533) were incubated for 48 h in growth media (RPMI 1640 supplemented with 10% [vol/vol] heat-inactivated FBS, 50 μM 2-sulfanylethan-1-ol [both Sigma], 100 µM non-essential amino acids, 1 mM sodium pyruvate, 0.1 mg/mL streptomycin, 100 U/mL penicillin [all Gibco]) supplemented with 29 nM CCL19 (R&D Systems) before infection with 0.4 MOI (multiplicity of infection) HIV-1_NL4-3_ for 3 h. Cells were washed three times to remove cell-free virus, plated at 5 × 10^5^ cells/mL in fresh growth media supplemented with 250 ng/mL staphylococcal enterotoxin B (Sigma) and 25 U/mL IL-2 (Roche), and cultured for 3 d. Cells were then washed and resuspended at 5 × 10^5^ cells/mL in fresh growth media supplemented with 25 U/mL IL-2 and cultured for 12 d. Memory CD4+ T cells were enriched from this culture by negative selection using a memory CD4+ T cell isolation kit (Miltenyi Biotec) and cultured in basal media (RPMI 1640 supplemented with 10% (vol/vol) heat-inactivated FBS, 0.1 mg/mL streptomycin, 100 U/mL penicillin), supplemented with 1 ng/mL IL-7 (R&D Systems) for 30 d.

TZM-bl cells were cultured in DMEM supplemented with 10% (vol/vol) heat-inactivated FBS, 0.1 mg/mL streptomycin, and 100 U/mL penicillin. SUP-T1 cells (human; sex: male) (ATCC Cat# CRL-1942, RRID:CVCL_1714) were cultured in RPMI 1640 supplemented with 10% (vol/vol) heat-inactivated FBS, 0.1 mg/mL streptomycin, and 100 U/mL penicillin. All incubations were performed in 5% CO_2_ at 37 °C.

### HIV-1 neutralization assay

Neutralization activity of TNP was measured using pseudovirus in a luciferase-based assay in TZM-bl cells as previously described 40, 51. Briefly, serial dilutions of TNP were incubated with 200 TCID_50_ virus in presence of diethylaminoethyl-dextran and the neutralizing activity was assessed by measuring luciferase activity after 48 h using a FilterMax F5 multi-mode microplate reader (Molecular Devices). Dose-response curves were fitted using nonlinear regression to determine IC_50_ and IC_80_ values (Prism v. 8, GraphPad).

### Cytotoxicity and cell viability

Cell death was estimated by staining cells for HIV-1 antigens and exposed phosphatidylserine using anti-phosphatidylserine (Millipore Cat# 05-719, RRID:AB_309933), anti-HIV-1 p55+p24+p17 (Bioss Cat# BS-4942R, RRID:AB_2892128), Alexa Fluor 488 (AF488) conjugated goat anti-rabbit IgG (Cell Signaling Technology Cat# 4412, RRID:AB_1904025), and phycoerythrin (PE) conjugated goat anti-mouse IgG (Cell Signaling Technology Cat# 8887, RRID:AB_2797678) using a procedure as previously described 11. Flow cytometry acquisition was carried out on a FACSCalibur flow cytometer followed by analysis using CellQuest Pro software (both BD Biosciences​). Lactate dehydrogenase (LDH) activity of supernatants was measured using a mixture of diaphorase/NAD^+^ and iodonitrotetrazolium chloride/sodium 2-hydroxypropanoate and % cytotoxicity calculated according to the manufacturer's protocol (Takara Bio).

### siRNA transfection

Cells were transfected with Silencer Select ATG5 (ID# s18159) or ATG7 (ID# s20651) or control (Cat# 4390846) siRNA (siNS) using lipofectamine RNAiMAX transfection reagent (all Invitrogen) in Opti-MEM (Gibco) according to the manufacturer's instructions. 48 h later, cells were analyzed for target gene silencing and used in experiments. Transfection efficiency was assessed with BLOCK-iT Alexa Fluor Red Fluorescent Control (Invitrogen) using flow cytometry 10.

### Western blotting

The following antibodies were used: CCR5 (Cat# ab65850, RRID:AB_1140936), CXCR4 (Cat# ab124824, RRID:AB_10975635), and SQSTM1 (Cat# ab56416, RRID:AB_945626) from Abcam, CD4 (Cat# 300501, RRID:AB_314069) from BioLegend, ATG5 (Cat# 2630, RRID:AB_2062340), ATG7 (Cat# 2631, RRID:AB_2227783), BIRC2 (Cat# 7065, RRID:AB_10890862), CASP8 (Cat# 9746, RRID:AB_2275120), PARP1 (Cat# 9532, RRID:AB_659884), RIPK1 (Cat# 3493, RRID:AB_2305314), XIAP (Cat# 2045, RRID:AB_2214866) from Cell Signaling Technologies, MAP1LC3B (Cat# NB100-2220) from Novus Biologicals, and ACTB (Cat# A2228, RRID:AB_476697) from Sigma. Whole cell lysates and TNP membranes were prepared, resolved, and proteins detected as previously described 10, 52. Relative densities of the target bands were compared to the reference (ACTB) and were calculated using Fiji from the Max Planck Institute of Molecular Cell Biology and Genetics (Fiji, RRID:SCR_002285) 53.

### Statistics

Samples were assigned to experimental groups through simple random sampling. Sample size was determined using a 2-sample 2-sided equality test with power (1-*β*) = 0.8, *α* = 0.05 and preliminary data where the minimum difference in outcome was at least 70%. Sample sizes are denoted in figure legends [n]. Data are represented as dot blots with arithmetic means ± 95% confidence interval for independent biological replicates, and as the arithmetic mean ± standard deviation for technical replicates. Data were assessed for symmetry, or skewness, using Pearson's skewness coefficient. Normalized ratiometric data were log_2_ transformed. Comparisons between groups were performed using the paired, two-tailed, Student's *t* test. *P* values were determined on the basis of biological replicates (with technical replicates averaged within each biological replicate). In all experiments, differences were considered significant when *P* was less than 0.05. *P < 0.05.

## Results

### Characterization of the TNP

**Figure 1:**
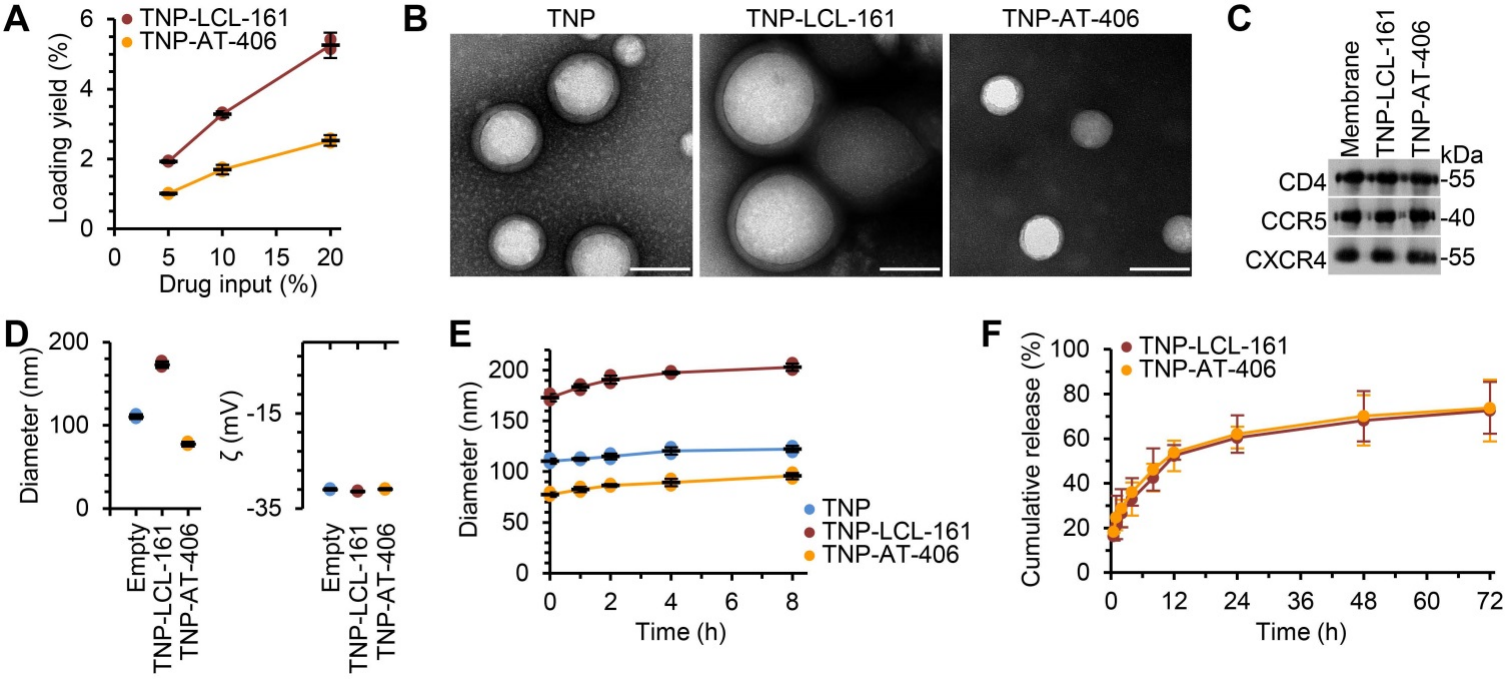
** Physicochemical characterization of DIABLO/SMAC mimetic loaded TNP (SM-TNP). (A)** Encapsulation efficiency of DIABLO mimetics (LCL-161 and AT-406) into TNP at 5%, 10%, and 20% initial drug input. *n* = 3. **(B)** Transmission electron microscopy images of TNP formulations negatively stained with uranyl acetate. Scale bar = 100 nm. **(C)** Western blots probing for CCR5, CD4, and CXCR4 in purified CD4+ T cell membrane fractions and SM-TNP formulations. **(D)** *Left*, hydrodynamic size of TNP formulations (diameter, nm). *Right*, surface zeta potential of TNP formulations (ζ, mV). *n* = 3. **(E)** Stability of TNP formulations in PBS determined by size measurement (diameter, nm) over the course of 8 h. *n* = 3. **(F)** Cumulative release profile of DIABLO mimetics from SM-TNP formulations over 72 h. *n* = 3.

To improve the bioavailability and specificity of the DIABLO mimetics, we encapsulated the drugs into TNP. The nanoformulation consisted of a DIABLO mimetic-loaded PLGA core, to which we fused the plasma membranes of uninfected CD4+ T cells as described previously 39. Drug loading yield increased as the drug input concentration for both LCL-161 and AT-406 was increased (Figure 1A); maximal loading was achieved at 20 wt% drug input before the nanoparticles became unstable, and this formulation was used in subsequent studies. Under examination by transmission electron microscopy, the TNP showed a typical core-shell structure depicting a uniform membrane coating around the core (Figure 1B). We then confirmed that key surface antigens, including CD4, C-X-C motif chemokine receptor 4 (CXCR4), and C-C motif chemokine receptor 5 (CCR5) were translocated with the CD4+ T cell membranes onto the polymeric cores (Figure 1C). Dynamic light scattering (DLS) measurements showed that, upon LCL-161 encapsulation, the diameter of the nanoparticles increased from 110.3 ± 2.5 to 173.0 ± 3.6 nm, but the surface zeta potential remained unchanged, further confirming complete CD4+ T cell membrane coating (Figure 1D). Conversely, encapsulation of AT-406 resulted in a decrease of nanoparticle size to 77.3 ± 2.3 nm, again with no change in surface zeta potential. The variation in the final size of the nanoformulations likely resulted from differences in how the drug payload interacted with the PLGA matrix during the self-assembly process. The TNP also showed a high colloidal stability over 8 h, with all three TNP maintaining a stable size (Figure 1E). In terms of the drug release kinetics, initial burst release was observed within 1 h, and 50% of both AT-406 and LCL-161 was released by 12 h (Figure 1F). Collectively, these data indicate the successful fabrication of DIABLO mimetic polymeric cores with CD4+ T cell membranes.

### TNP broadly neutralize Env-pseudotyped HIV-1

To assess the breadth and potency of the TNP to neutralize HIV-1, we used the global 12-virus panel 50 with a validated neutralization protocol 40, 54, 55 (Table 1). Against this panel, we observed that the neutralization breadth of the loaded TNP was 100%. The neutralization potency (geomean 50% inhibitory concentration [IC_50_]/IC_80_) of the loaded TNP was robust against all 12 viruses, with the IC_50_/IC_80_ lower in both the TNP-LCL-161 (66.95/288.14 µg/mL) and the TNP-AT-406 (53.84/238.92 µg/mL) than in the empty TNP (182.74/1030.74 µg/mL) with no subtype preference evident.

### DIABLO/SMAC mimetic-loaded TNP selectively kill HIV-1-infected cells through an autophagy-dependent mechanism

**Figure 2:**
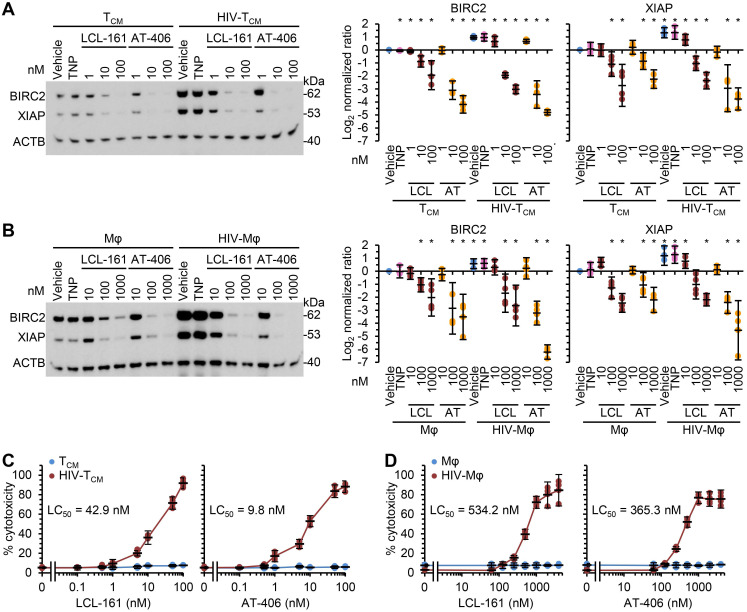
** DIABLO/SMAC mimetic loaded TNP preferentially induce cell death in HIV-1-infected cells.** Mock- and HIV-1-infected T_CM_ **(A, C)** or macrophages** (B, D)** were exposed to vehicle, empty TNP (TNP) or DIABLO/SMAC mimetic loaded TNP (LCL-161/LCL or AT-406/AT) for 4 h, washed three times with PBS, and then incubated for a further 24 h in fresh media. *n* = 4. **(A, B)** *Left*, representative western blots of BIRC2, XIAP and ACTB. *Right*, densitometric analysis of blots. **(C, D)** Aliquots of supernatants were spectrophotometrically tested for LDH as a measure of cell death.

As SM induce the rapid degradation of BIRC2 and XIAP, a key early event in SM-induced cell death 10, 12, 19, 20, 56, we evaluated the ability of the DIABLO/SMAC mimetic loaded TNP (SM-TNP) to induce BIRC2 and XIAP degradation in HIV-Mφ, HIV-T_CM_, and their uninfected counterparts. Whereas the empty TNP had no effect on the expression of BIRC2 or XIAP, both SM-TNP induced significant BIRC2 and XIAP degradation in both uninfected cells and HIV-1-infected cells (Figure 2A and 2B). However, the concentrations required to induce significant degradation were 10-100 × lower in HIV-1-infected cells than in uninfected cells.

**Figure 3:**
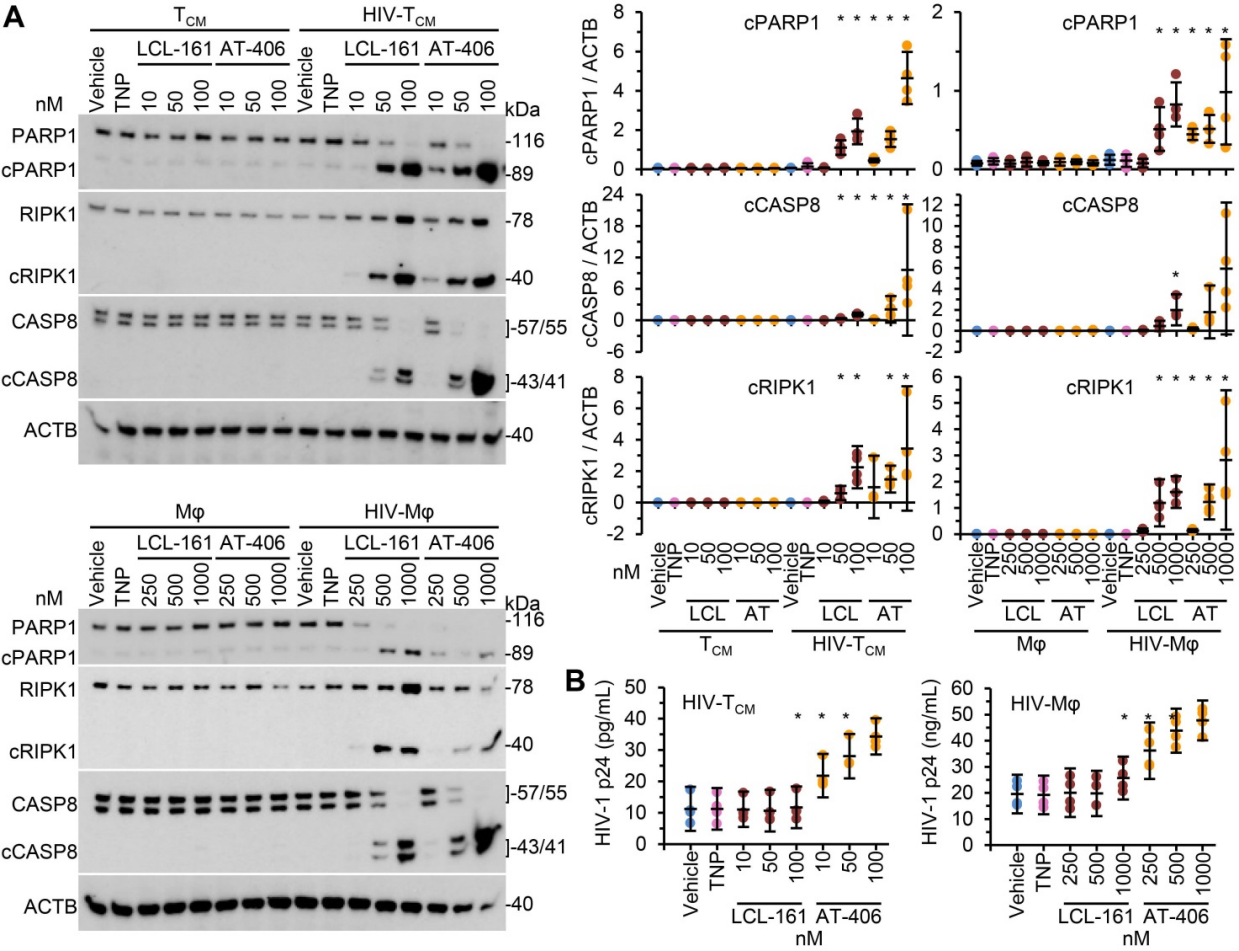
** DIABLO/SMAC mimetic loaded TNP induce PARP1 cleavage in HIV-1-infected cells.** Mock- and HIV-1-infected T_CM_ or macrophages (Mφ) were exposed to vehicle, empty TNP (TNP) or DIABLO/SMAC mimetic loaded TNP (LCL-161/LCL or AT-406/AT) for 4 h, washed three times with PBS, and then incubated for a further 24 h in fresh media. *n* = 4. **(A)** *Left*, representative western blots of PARP1, cleaved PARP1 (cPARP1), RIPK1, cleaved RIPK1 (cRIPK1), caspase 8 (CASP8) and β-actin (ACTB). *Right, d*ensitometric analysis of blots is shown. **(B)** Aliquots of supernatants were tested for HIV-1 p24 antigen by ELISA.

We next evaluated the toxicity of the SM-TNP against both uninfected and infected cells using the release of lactate dehydrogenase as a marker of cell death. In uninfected T_CM_ and macrophages, both TNP-LCL-161 and TNP-AT-406 showed minimal toxicity at all concentrations tested (Figure 2C and 2D). Conversely, both induced a dose-dependent increase in HIV-T_CM_ and HIV-Mφ cytotoxicity, with the concentrations required to induce cell death being 10× greater in HIV-Mφ than in HIV-T_CM_. This increase in cytotoxicity in infected cells corresponded to the dose-dependent proteolysis of poly(ADP-ribose) polymerase 1 (PARP1; a substrate of caspase [CASP] 3), CASP8 and RIPK1 cleavage (Figure 3A). Importantly, we measured no significant TNP-LCL-161-mediated increase in HIV-1 p24 antigen release from either HIV-T_CM_ or HIV-Mφ indicating that the TNP-LCL-161 kills HIV-1-infected cells in the absence of increased virus production (Figure 3B). Conversely, TNP-AT-406 treatment induced a dose-dependent increase in HIV-1 p24 antigen release from both HIV-T_CM_ and HIV-Mφ, highlighting its previously described potential as a latency reversal candidate 57 (Figure 3B).

**Figure 4:**
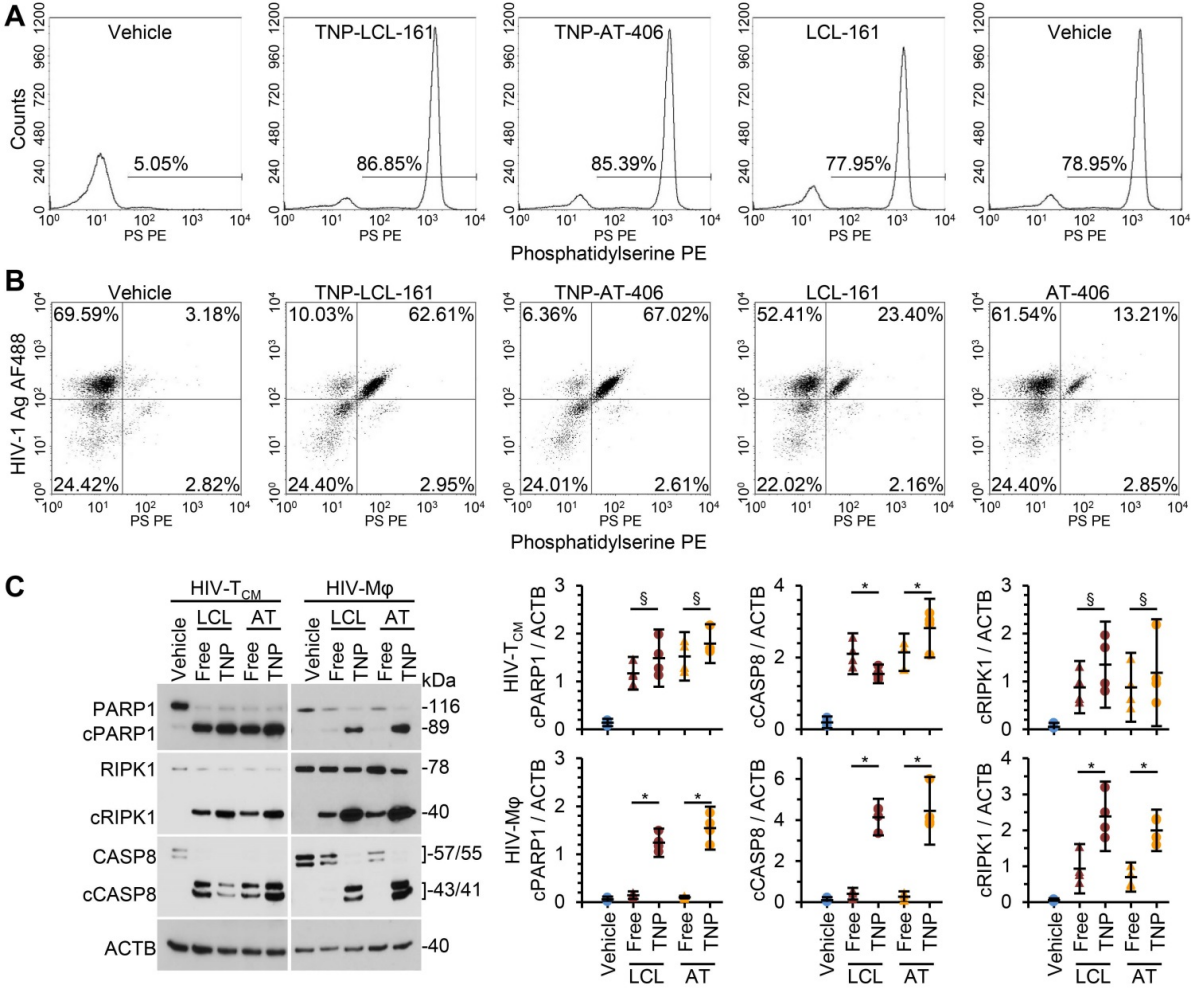
** DIABLO/SMAC mimetic loaded TNP are more effective than free drug at killing HIV-1-infected cells.** HIV-1-infected T_CM_ (HIV-T_CM_) or macrophages (HIV-Mφ) were exposed to vehicle, DIABLO/SMAC mimetic loaded TNP (LCL-161/LCL or AT-406/AT), or free drug for 4 h, washed three times with PBS, and then incubated for a further 24 h in fresh media. HIV-T_CM_ were treated with 100 nM of each drug/TNP and HIV-Mφ with 1 µM drug/TNP. *n* = 4. **(A)** HIV-T_CM_ were surface stained for exposed phosphatidylserine (Phosphatidylserine PS [phycoerythrin]), fixed, washed then harvested and analyzed by flow cytometry. A representative donor is shown. **(B)** HIV-Mφ were surface stained for exposed phosphatidylserine, fixed, permeabilized, then stained for HIV-1 p55+p24+p17 (HIV-1 Ag AF488 [Alexa Fluor 488]), washed, then harvested and analyzed by flow cytometry. A representative donor is shown. **(C)** *Left*, representative western blots of PARP1, cleaved PARP1 (cPARP1), RIPK1, cleaved RIPK1 (cRIPK1), caspase 8 (CASP8) and β-actin (ACTB). *Right, d*ensitometric analysis of blots is shown.

We next compared the toxicity of the SM-TNP against free drug. In HIV-T_CM_, at the doses and times tested, the SM-TNP induced similar levels of phosphatidylserine exposure as free SM (Figure 4A). Conversely, in HIV-Mφ the SM-TNP induced significantly more phosphatidylserine exposure on cells expressing HIV-1 antigens than on those not expressing HIV-1 antigens (Figure 4B). These similarities and differences were mirrored in the cleavage of PARP1, CASP8, and RIPK1. While we observed no significant difference in PARP1 or RIPK1 cleavage between SM-TNP treatment and free SM treatment of HIV-T_CM_, we observed significant differences in HIV-Mφ, with the SM-TNP inducing significantly more PARP1 and RIPK1 cleavage than the free SM (Figure 4C). Importantly, the results of free drug treatment are similar to those already published 12.

**Figure 5:**
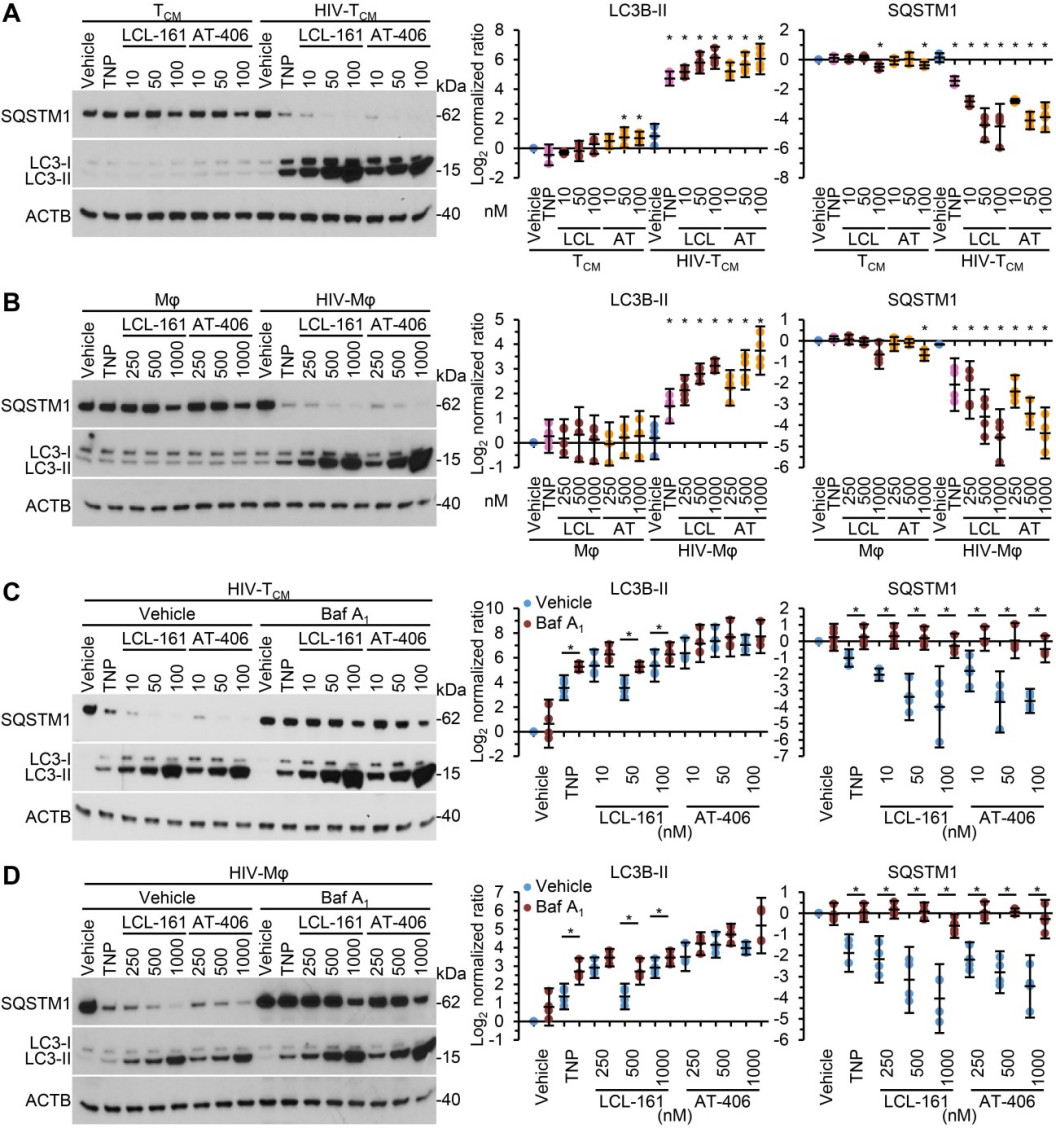
** DIABLO/SMAC mimetic loaded TNP induce autophagy. (A, B)** Mock- and HIV-1-infected T_CM_ (A) or macrophages (Mφ) (B) were exposed to vehicle, empty TNP (TNP) or DIABLO/SMAC mimetic loaded TNP (LCL-161/LCL or AT-406/AT) for 4 h, washed three times with PBS, and then incubated for a further 24 h in fresh media. *Left*, representative western blots of LC3B isoforms and SQSTM1. *Right*, densitometric analysis of blots. *n* = 4. **(C, D)** Mock- and HIV-1-infected T_CM_ (C) or macrophages (D) were pre-treated with bafilomycin A_1_ (Baf A_1_) for 1 h before exposed to vehicle, empty TNP (TNP) or DIABLO/SMAC mimetic loaded TNP (LCL-161/LCL or AT-406/AT) for 4 h, washed three times with PBS, and then incubated for a further 24 h in fresh media. *Left*, representative western blots of LC3B isoforms and SQSTM1. *Right*, densitometric analysis of blots and LDH assay. *n* = 4.

As SM-mediated cell death of HIV-1-infected cells is dependent upon the induction of autophagy 10, 12 we next assessed whether the TNP-SM induce autophagy. Autophagic flux is assessed by monitoring the biogenesis of autophagosomes and the degradation of their contents. During autophagy, a ubiquitin-like system that involves autophagy related (ATG) 7 and the ATG12-ATG5 complex converts cytosolic microtubule-associated protein 1 light chain 3 beta (MAP1LC3B or LC3B)-I to LC3B-II and ligates it to the nascent isolation membrane and SQSTM1 binds to LC3B-II. After detachment of ATG factors, the isolation membrane closes and syntaxin 17 is recruited to the autophagosome. These then fuse with lysosomes resulting in the degradation of the autophagosome contents, including the intraluminal LC3B-II and SQSTM1. Thus, the quantification and turnover of SQSTM1 and LC3B-II in a lysosome-dependent manner are indicators of autophagic flux 58. In uninfected macrophages and T_CM_, SM-TNP increased LC3B lipidation at the highest concentrations tested while having no significant effect on SQSTM1 degradation indicating an absence of induced autophagy (Figure 5A and 5B). In contrast, both TNP-LCL-161 and TNP-AT-406 induced a dose-dependent significant increase in LC3B-II lipidation and SQSTM1 degradation in HIV-T_CM_ and HIV-Mφ. To confirm the induction of autophagic flux in TNP-SM-treated HIV-1-infected cells we used bafilomycin A_1_, an inhibitor of autophagosome-lysosome fusion. Blots of cell lysates confirmed autophagic flux in both HIV-T_CM_ and HIV-Mφ, with increased LC3B-II and SQSTM1 accumulation in bafilomycin A_1_ treated cells relative to vehicle controls (Figure 5C and 5D).

**Figure 6:**
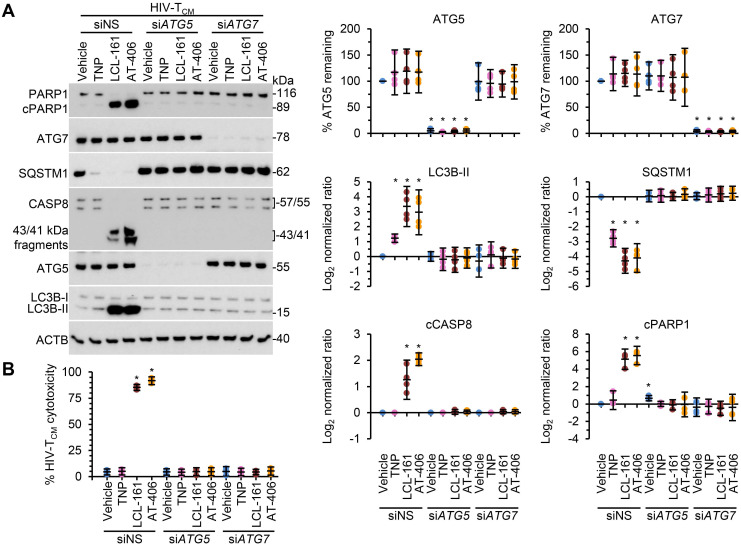
** DIABLO/SMAC mimetic loaded TNP induce autophagy-dependent apoptosis of HIV-1-infected T_CM_.** HIV-1-infected T_CM_ (HIV-T_CM_) transduced with *ATG5* siRNA (siATG5), *ATG7* siRNA (si*ATG7*), or scrambled siRNA (siNS) were exposed to vehicle, empty TNP (TNP) or DIABLO/SMAC mimetic loaded TNP (LCL-161 or AT-406 at 100 nM) for 4 h. Cells were then washed three times with PBS, and then incubated for a further 24 h in fresh media. **(A)** *Left*, representative western blots of ATG5 (ATG5-ATG12), ATG7, CASP8, cleaved CASP8 (cCASP), LC3B isoforms, PARP1, cleaved PARP1 (cPARP1), SQSTM1, and β-actin (ACTB). *Right*, densitometric analysis of blots. **(B)** Aliquots of supernatants were spectrophotometrically tested for LDH as a measure of cell death.

**Figure 7:**
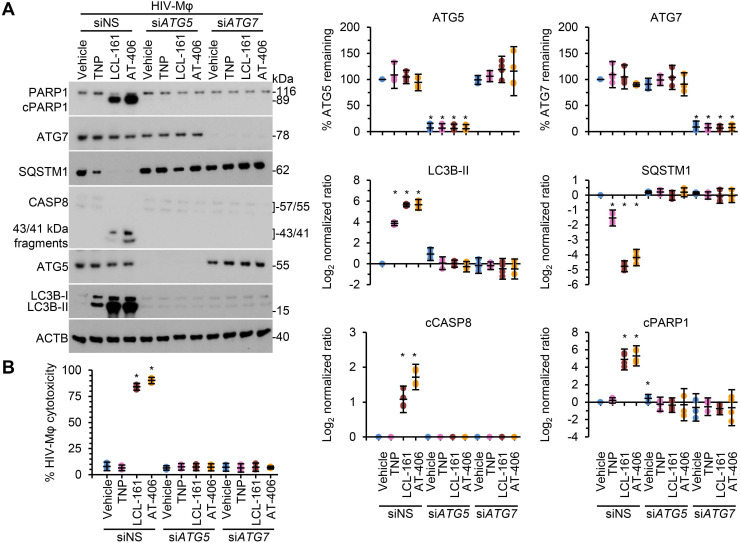
** DIABLO/SMAC mimetic loaded TNP induce autophagy-dependent apoptosis of HIV-1-infected macrophages.** HIV-1-infected macrophages (HIV-Mφ) transduced with *ATG5* siRNA (siATG5), *ATG7* siRNA (si*ATG7*), or scrambled siRNA (siNS) were exposed to vehicle, empty TNP (TNP) or DIABLO/SMAC mimetic loaded TNP (LCL-161 or AT-406 at 1 µM) for 4 h. Cells were then washed three times with PBS, and then incubated for a further 24 h in fresh media. **(A)** *Left*, representative western blots of ATG5 (ATG5-ATG12), ATG7, CASP8, cleaved CASP8 (cCASP), LC3B isoforms, PARP1, cleaved PARP1 (cPARP1), SQSTM1, and β-actin (ACTB). *Right*, densitometric analysis of blots. **(B)** Aliquots of supernatants were spectrophotometrically tested for LDH as a measure of cell death.

Following the SM-mediated degradation of BIRC2 and XIAP and the induction of autophagy in HIV-1-infected cells, a death inducing signaling complex is formed on phagophore membranes that includes both pro-apoptotic or necroptotic (FADD, RIPK1, RIPK3, caspase 8, MLKL) and autophagy (ATG5, ATG7 and SQSTM1) proteins. Disruption of this complex using RNA interference (RNAi) for either *ATG5* or *ATG7* inhibits the formation of the death inducing signaling complex and thus SM-mediated apoptosis 10, 12, 59. Therefore, we next assessed whether autophagy was essential for TNP-SM-mediated apoptosis of HIV-1-infected cells by employing RNAi for *ATG5* and *ATG7*. In these experiments, inhibition of the autophagy conjugation completely ablated the cell death response to TNP-SM in both HIV-T_CM_ (Figure 6) and HIV-Mφ (Figure 7) indicating that the mechanism of cell death is autophagy-dependent.

## Discussion

Currently available ART has greatly improved life expectancy and quality for those infected with HIV-1. However, an effective vaccine is still elusive, and multi-drug resistance continues to increase. Over the last decade, fundamental computational biology and clinical research into the “kick and kill” strategy have highlighted the complexity and highly heterogeneous nature of HIV-1 proviral reservoirs. Although this research has led to several key developments, such as HIV-1-specific broadly neutralizing antibody (HIV-1-bnAb) and the application of HIV-1-specific natural killer cells, the research has also cast doubt on this strategy as limitations include the failure to induce expression from the entire HIV-1 latent reservoir, the inability to ensure complete limitation of viral rebound, and the absence of latent HIV-1-infected cell death upon reactivation 1. Additionally, HIV-1-bnAb are ineffective against all circulating strains and are subject to poor efficacy and viral escape 60, 61 emphasizing the importance of identifying alternative strategies to complement existing options. To this end, we developed the TNP to enable targeting of HIV-1-infected cells 39, 40. The manufacturing and application of TNP does not require prior mapping and optimization of epitopes, nor is it dependent upon quasispecies sequencing or drug resistance profiling. Instead, the TNP are assembled using biomimetic nanotechnology that mimics natural CD4+ T cells to bind to and neutralize HIV-1. We previously showed that unloaded TNP show excellent binding to HIV-1 gp120, and neutralize all 125 strains in a global panel while also reducing cell-associated HIV-1 in the absence of cell death 40. Thus, by leveraging the natural binding affinity of CD4+ T cell membrane receptors to HIV-1 gp120, we have overcome the high glycosylation, steric restriction, and rapid mutation rate of gp120 that limits HIV-1-bnAb development and which leads to poor HIV-1-bnAb efficacy and viral escape, as although HIV-1 gp120 can mutate to become antibody resistant, it must retain its ability to bind CD4 to infect cells.

In the present study, we loaded TNP with DIABLO/SMAC mimetics and show that these loaded TNP not only neutralize cell-free HIV-1, but also selectively kill HIV-1-infected cells through autophagy-dependent apoptosis 10, 12. The SM-mediated degradation of IAPs in HIV-1-infected cells leads to the ATG5- and SQSTM1-dependent translocation and interaction of FADD with the CASP8 homocomplex, RIPK1, and RIPK3, and the formation of a cytosolic death-inducing signaling complex on autophagosome initiation membranes, thus linking autophagy to the control of cell survival and apoptosis; a process that does not occur in SM-treated uninfected cells 10, 12. Importantly, TNP-LCL-161 failed to significantly increase viral production from either HIV-T_CM_ or HIV-Mφ. These findings align with previous studies that demonstrated that LCL-161 failed to activate latent provirus in resting CD4+ T cells collected from HIV-1-infected patients undergoing ART or from HIV-Mφ 12, 16. Conversely, we observed that TNP-AT-406 increased the production of HIV-1 p24 from both HIV-T_CM_ and HIV-Mφ in agreement with previous studies 12, 57. Significantly, the encapsulation of SM within TNP decreased the time and SM concentration required to kill infected cells. In addition to encapsulating SM within TNP, additional payloads can be loaded into the membrane bilayers, opening additional potential mechanisms of viral suppression and/or selective killing of infected cells as well as anatomical site targeting.

One of the major tasks in HIV-1 cure research is to eliminate infected cells that are disseminated broadly across numerous tissues, including sites that may be relatively inaccessible to host defenses or treatment strategies such as the lymphatic tissue and the central nervous system where concentrations of antiviral drugs are lower than in peripheral blood 62, 63. Under certain conditions, nanoparticles are able to enter both the lymphatic tissue and the central nervous system 64, 65, which may enhance both their neutralization activity and inhibition of HIV-1 replication. Therefore, future research will need to define the optimal TNP characteristics required for ideal accumulation in lymph nodes and the central nervous system, combined with other properties to balance the TNP pharmacokinetic profile and viral binding efficiency *in vivo* for maximum outcome.

With substantially better breadth, potency and resistance to viral escape than HIV-1-bnAb, TNP are a promising candidate for *in vivo* development. Unlike current antiviral treatments that target the virus itself, the development of resistance to SM-TNP is unlikely as this strategy minimizes viral resistance by working at the host level to kill HIV-1-infected cells, while escape is unexpected as the TNP exploit the requirement of HIV-1 to bind CD4 on target cells. Additionally, TNP loaded with SMAC/DIABLO mimetics or other cytotoxic drugs could be used as part of initial treatment of persons infected with HIV-1. In this scenario, SM-TNP combined with ART and/or second generation MTOR inhibitors could suppress viral replication while preferentially killing HIV-1-infected cells, thus contributing to a cure strategy. In summary, TNP represent a promising drug delivery platform to both neutralize HIV-1 and to deliver cytotoxic agents specifically to HIV-1-infected cells while minimizing off-target and cytotoxic effects on bystander cells.

## Figures and Tables

**Table 1:** Neutralization of HIV-1 Env-pseudoviruses across major circulating clades by TNP

		Subtype and strain												GeoMean	%
		A	A	B	B	B	C	C	CRF_01	CRF_01	CRF_07	CRF_07	G		
		246F3	398_F1	X2278	TRO.11	CE1176	CE703	25710	CNE55	CNE8	BJOX002	CH119	X1632		
Empty TNP	IC_50_	261.20	244.10	365.60	161.58	22.11	122.30	202.50	169.90	802.95	183.70	227.40	118.00	182.74	100
	IC_80_	1634.40	1126.00	1887.00	757.20	2560.00	582.80	989.40	696.80	1265.65	744.80	861.90	654.40	1030.74	100
TNP-LCL-161	IC_50_	92.43	60.92	143.30	51.81	125.40	27.38	86.25	34.38	24.68	49.73	55.80	278.30	66.95	100
	IC_80_	911.60	171.20	471.60	95.80	478.60	248.00	504.30	141.70	151.60	246.90	111.00	1318.00	288.14	100
TNP-AT-406	IC_50_	85.51	31.50	47.59	38.66	91.60	80.25	70.55	28.89	38.91	31.77	135.90	47.58	53.84	100
	IC_80_	456.30	105.40	180.90	192.00	325.50	104.40	431.70	209.40	251.80	150.90	744.90	238.20	238.92	100

Neutralization potency (IC_50_/IC_80_) expressed as *µ*g/mL and neutralization breadth (expressed as a percentage; %) of the TNP against different HIV-1 Env-pseudoviruses.

## Abbreviations

ART: antiretroviral therapy
ATG: autophagy related
BIRC: baculoviral IAP repeat containing
CASP: caspase
CCR5: C-C motif chemokine receptor 5
CXCR4: C-X-C motif chemokine receptor 4
DIABLO: diablo IAP-binding mitochondrial protein
FBS: fetal bovine serum
HIV-1: human immunodeficiency virus type 1
HIV-1-bnAb: HIV-1-specific broadly neutralizing antibody
HIV-Mφ: HIV-1-infected macrophages
HIV-TCM: HIV-1-infected resting memory CD4+ T cells
IAP: inhibitor of apoptosis protein
IC_50_: 50% inhibitory concentration
IC_80_: 80% inhibitory concentration
IL: interleukin
LC3: microtubule-associated protein 1 light chain 3 beta
LDH: lactate dehydrogenase
LRA: latency-reversing agents
MAP1LC3B: microtubule-associated protein 1 light chain 3 beta
MOI: multiplicity of infection
PBS: phosphate-buffered saline
RIPK: receptor-interacting protein kinase
siNS: scrambled control siRNA
siRNA: small interfering RNA
PARP1: poly(ADP-ribose) polymerase 1
PBMC: peripheral blood mononuclear cells
PE: phycoerythrin
PLGA: poly(lactic‐*co*‐glycolic acid)
SM: DIABLO/SMAC peptidomimetics
SM-TNP: TNP encapsulated DIABLO/SMAC peptidomimetics
SMAC: second mitochondria-derived activator of caspase
SQSTM1: sequestosome 1
TCID50: 50% tissue culture infectious doses
TNP: CD4+ T cell membrane-coated nanoparticles
XIAP: X-linked inhibitor of apoptosis
